# Based case based learning and flipped classroom as a means to improve international students’ active learning and critical thinking ability

**DOI:** 10.1186/s12909-024-05758-8

**Published:** 2024-07-15

**Authors:** Wanjing Yang, Xiaoyan Zhang, Xinhuan Chen, Jing Lu, Fang Tian

**Affiliations:** 1 Department of Pathophysiology, School of Basic Medical Sciences, Zhengzhou University, Zhengzhou, 450001 China; 2 Department of Pathology and Forensic Medicine, School of Basic Medical Sciences, Zhengzhou University, Zhengzhou, 450001 China

**Keywords:** Case-based learning, Flipped classroom, Active learning, Critical thinking, International students

## Abstract

**Background:**

International student education has become an important part of higher education and an important symbol to measure the level of higher education. To change the traditional teaching model, here we introduced a combination of Case-Based Learning (CBL)and Flipped Classroom (FC) into the pathophysiology course for international students. This study aimed to explore whether the active learning ability and critical thinking ability of international students can be improved, based on this new teaching model, improving the innovation ability of teachers’ team and students’ attitude to the reform.

**Methods:**

The two chapters of Cardiac Insufficiency and Apoptosis in Pathophysiology are designed as a CBL + FC teaching method. Distribute the Self-assessment Scale on Active Learning and Critical Thinking (SSACT) and satisfaction questionnaire to international students to evaluate teaching reform based on CBL + FC.

**Results:**

Compared with the traditional classroom, the online flipped classroom based on CBL has significantly improved the learning enthusiasm, as these students are required to independently complete literature review, actively participate in classroom teaching, learn to use multiple learning strategies, and collaborate with other students to complete PowerPoint (PPT)production. At the same time, the students’ ability to raise problems and solve problems has been greatly improved by analyzing clinical cases; By consulting the literature, the theoretical knowledge learned can be better applied to clinical analysis. The results of the satisfaction survey also show that international students are more likely to accept the flipped classroom teaching mode.

**Conclusions:**

This teaching mode will stimulate the learning motivation of international students, enhance teaching attraction and increase teaching interaction; At the same time, the CBL + FC teaching method can strengthen the evaluation of international students’ in and out of class and online learning, enhance students’ active learning ability and critical thinking ability, promote the development of personalized learning, and integrate with international medical education.

## Introduction

At the beginning of the new-year in 2020, the epidemic caused by covid-19 is a major global public health event. Among the people affected by these restrictions, there is a special group of international students who come to China’s colleges and universities to study abroad [1]. Through the practice of teaching and management of international students for many years, research shows a clear understanding of the characteristics of the group of international students, such as: (1) most of the group of international students come from Asian countries, with great regional cultural differences; (2) The basic education level and development level of each country are different, and the acceptance ability also varies greatly; (3) The vast majority of international students like to participate, like to communicate with teachers, interactive learning and group discussion teaching [2–4]. Therefore, according to the characteristics of international students, how to change the traditional teaching mode of Pathophysiology for international medical students online with the single output of teachers, insufficient learning initiative of international students, paying attention to the deep integration of modern information technology and education and teaching, enhancing the interaction between teachers and students, students and students, has become an urgent problem to solve.

Active learning and continuous quality improvement are critical strategies when designing and refining medical school curricula [5, 6]. Some studies showed that medical educators, both at our institution and nationwide, are deliberately re-evaluating their curricula to incorporate active learning instructional methodology [5, 7]. In the study of Rose et al. showed that active learning, with regular incorporation of student feedback vis-àvis a PDSA cycle, was effective in achieving high student engagement in an Internal Medicine core clerkship session on antibiotic therapy. The study findings have potential implications for medical education and suggest that the application of the PDSA cycle can optimize active learning pedagogies and outcomes [8].

Critical thinking (CT) plays a central role in one’s learning and working, particularly in addition to medical education that transitions from knowledge-based curricula to competency-based curricula. It is crucial for students to learn and work further critically to evaluate existing knowledge and information. CT is vital to a health professional’s competence to assess, diagnose and care for patients correctly and effectively [9]. Studies by Ramlaul et al. indicated that as participants progressed from year one to year three, they recognized that critical thinking comprised not only of cognitive skills but affective skills too. They attributed their developing understanding of the meaning of critical thinking to clinical placement learning, understanding written feedback, and the expectations of professional practice [10]. Several other studies also revealed the relationship between critical thinking and academic success of medical professionals, such as a students’ PBL performance, health professions education and the motivation for learning [11–14].

Blended learning is a comprehensive and three-dimensional teaching mode that integrates various means such as offline teaching, online learning resources utilization, and mutual evaluation and feedback between students and teachers and students. Therefore, in the course of curriculum construction, it is necessary to promote and popularize this teaching method on a large scale, so that all teachers and students can actively participate in an efficient operation of “teaching” and “learning”. The teacher will change from the leading role in the traditional classroom to the supporting role in the blended learning mode, becoming the leader and supervisor, while the students will change from the traditional supporting role to the leading role, becoming the center and active element of the teaching link. Through the implementation of mixed teaching, students can be trained to learn actively, and the former “I want to learn” has become the current “I want to learn” situation. In the mixed teaching mode, teachers have changed the traditional way of teaching students “what is” into heuristic teaching of teaching students “why”, and gradually cultivate students to develop critical thinking methods such as raising questions, solving problems, and questioning, thus greatly improving the efficiency of teaching and learning. Many classes in higher education institutes now employ blended learning; whereby students learn in part at a supervised face-to-face location on campus, and in part through the Internet with some elements of student choice over place and pace [15]. Of the many different models of blended learning in practice, the use of flipped classroom approach has become increasingly widespread [16–18].

More and more studies show that compared with traditional teaching methods, the teaching effect of flipped classroom does have certain advantages [19–21]. In addition, with the progress of COVID-19, the research of online flipped classroom has also made some progress. Hew et al. showed that the participants in the fully online flipped classes performed as effectively as participants in the conventional flipped learning classes. Their qualitative analyses of student and staff reflection data identify seven good practices for videoconferencing*-*assisted online flipped classrooms [22]. Students in flipped courses exhibited gains in critical thinking, with the largest objective gains in intermediate and upper-level courses. Results from this study suggest that implementing active-learning strategies in the flipped classroom may benefit critical thinking and provide initial evidence suggesting that underrepresented and first-year students may experience a greater benefit [23].

CBL is an established approach used across disciplines where students apply their knowledge to real-world scenarios, promoting higher levels of cognition. Thus the objective of this study is to explore whether the active learning ability and critical thinking ability of international students can be improved by applying the innovation ability of teachers’ team, updating teaching ideas, and using the reform of CBL + FC teaching mode, so as to lay a foundation for building a first-class course of Pathophysiology for international students.

## Materials and methods

### Research object

The study was conducted at the school of international education from 2021 to 2022, Zhengzhou University, which employs CBL + FC in the third grade international students (there are 296 students in total), majors include clinical medicine, pharmacy and stomatology. They were divided into two groups: 115 students majoring in clinical medicine participated in online flipped classrooms, while 181 students majoring in pharmacy and stomatology participated in traditional classrooms.

The same teaching faculty is responsible for the teaching process. Oral informed consent was obtained from each student and teacher. This study was approved by the school ethics committee (Zhengzhou University Life Sciences Ethics Review Committee).

### Curriculum implementation plan and method

The two chapters of Cardiac Insufficiency and Apoptosis are designed as a CBL + flipped classroom teaching method, specific implementation process: A case is divided into multiple scenarios (parts) and send to students one week before class. Students summarize the symptoms and signs of the patient from the case, propose possible diagnosis, discuss the mechanism and raise questions. It is important to ask questions from the case. These questions can be solved by searching online in time during discussion, while the deeper questions can be recorded and assigned to each student. Later, students can acquire knowledge and independence through self-study methods, such as reading books, checking materials and MOOC study. The team leader will summarize and refine the questions raised by each group member, and 4–5 students make them into PPT (6–7 min), and reported in the class; the team leaders can communicate with each other to avoid solving the same problems, and each group should have its own focus. At last, teachers conduct in-depth analysis of the case, and make a general comment on the contents of each reporting group, especially to find out the highlights and innovations of students in the reporting process, and timely encourage and praise them.

### Effect evaluation

the establishment and implementation of teaching information collection system, international students’ teaching evaluation system and information feedback system is not only an important teaching management measure, but also a way to mobilize students’ enthusiasm and give full play to their personality. At the same time, a questionnaire is designed to evaluate whether the teaching reform based on Case + flipped classroom can improve the autonomous learning ability and critical thinking ability of international students and whether they are satisfied with this teaching method.

### Limitations and design

When conducting intervention studies on international students, it is crucial to ensure the consistency of the baseline conditions as much as possible to reduce bias. Of course, there are also some confounding factors that can affect the experimental results, such as:

1) International students from different countries may have different study habits or personalities;

2) The academic performance of international students upon admission varies;

3) International students may have a stronger sense of self-awareness.

In response to these factors, the following measures have been taken:

1) To reduce the impact of these differences on the results, researchers randomly assigned international students from different countries and majors into two groups, rather than grouping them by country or major;

2) The educational reform was placed in the sixth semester, at which point international students have generally adapted to the teaching methods in China and their academic performance is relatively stable, so this educational reform mainly focused on the students’ learning attitudes rather than solely on their grades;

3) Given the strong sense of autonomy among international students, their own choices were fully respected on the premise of random grouping. At the same time, the impact was reduced through the use of anonymous surveys and ensuring privacy protection.

### Instruments for Self-assessment Scale on Active Learning and Critical Thinking (SSACT) and satisfaction questionnaire disposition assessment

The 14 item SSACT consisted of two domains “active learning” and “critical thinking” [24, 25]. A five-point Likert scale questionnaire was used to evaluate student before and after class, ranging from 1 (strongly disagree) to 5 (strongly agree) (Table 1).

A five-point Likert scale Satisfaction questionnaire with 17 items was used to evaluate student perceptions of the effectiveness of CBL + Fipped classroom when we finished the class, ranging from 1 (strongly disagree) to 5 (strongly agree) [26].

Students were allowed to finish the questionnaire via an internet website,

where the data were collected from their submission. There are 296 students in total, 278 of them took part in the survey. In addition,106 students participated in the online flipped classroom and 172 students participated in the traditional classroom.

**Table 1:** The final version of Self-Assessment Scale on active learning and critical thinking (SSACT)

No	Items						Factors/Subscales
1	I set my own learning objectives for each scenario, in addition to the group objectives.	5	4	3	2	1	Active Learning
2	I applied various learning strategies during independent study.	5	4	3	2	1	
3	I was able to summarize the key points of the outcome of the group discussion.	5	4	3	2	1	
4	I managed my independent study effectively.	5	4	3	2	1	
5	My behaviour encouraged other members to actively participate in the tutorial process.	5	4	3	2	1	
6	I reflected on my learning in each scenario based on the objectives that I set myself.	5	4	3	2	1	
7	I was able to formulate questions based on the scenario.	5	4	3	2	1	Critical Thinking
8	I communicated my ideas clearly.	5	4	3	2	1	
9	I performed the role given to me by other group members.	5	4	3	2	1	
10	In the second meeting, I applied knowledge from my independent study to provide a solution to the problem being discussed.	5	4	3	2	1	
11	I analysed information in the scenario using relevant theory and concepts.	5	4	3	2	1	
12	I made links during the tutorial process between my newly acquired knowledge and my previous knowledge.	5	4	3	2	1	
13	I explained knowledge from the resources in my own words.	5	4	3	2	1	
14	I could generate a hypothesis to explain the problem under discussion.	5	4	3	2	1	

Directions: Please read each statement and circle the number that best describes your thoughts and feelings about your own learning. There is no right or wrong answer

5 = Strongly agree, 4 = Agree, 3 = Neutral, 2 = Disagree, 1 = Strongly disagree

### Statistical analysis

SPSS software is used to analyze the scores of students’ creative thinking evaluation, critical thinking evaluation and satisfaction evaluation of online flipped classroom. The measurement data are expressed by means and standard deviation, T-test was used to statistically infer students’ creative thinking and critical thinking ability.

## Results

**Fig. 1:**
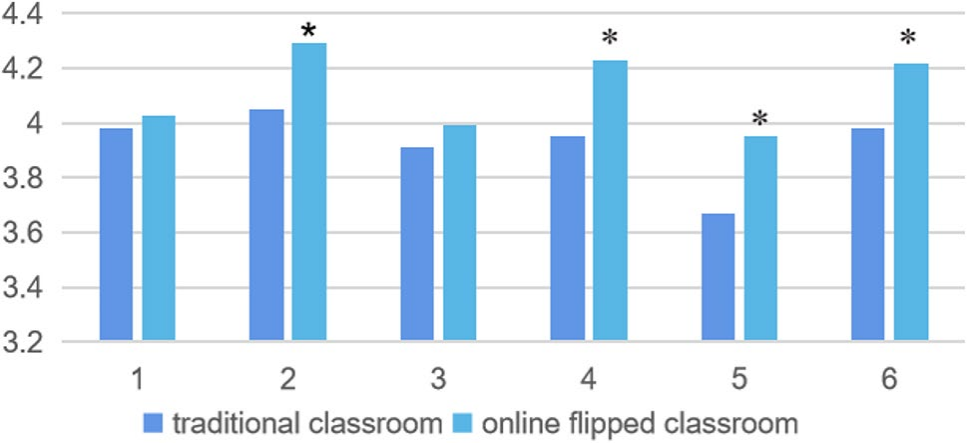
The comparison of active learning between online flipped classroom and traditional classroom. *Notes* *p < 0.05

1. In terms of students’ active learning, compared with the traditional classroom, the online flipped classroom has improved the basic learning enthusiasm (Fig. 1), especially in promoting the use of multiple learning strategies in autonomous learning (4.29 ± 0.74,*p* < 0.05), managed independent study effectively(4.23 ± 0.86, *p* < 0.05), encouraging other members to assist in learning (3.95 ± 1.01, *p* < 0.05), and reflected on the learning in each scenario based on the objectives (4.22 ± 0.86, *p* < 0.05). However, some students showed deficiencies in summarizing the key points of the outcome of the group discussion (Table 2).

**Table 2:** Comparison of active learning between online flipped classroom (*n* = 106) and traditional classroom (*n* = 172)

No	Items	Traditional classroom mean ± SD	Flipped classroom mean ± SD	*p* value
1	I set my own learning objectives for each scenario, in addition to the group objectives.	3.98 ± 0.89	4.03 ± 0.90	0.678
2	I applied various learning strategies during independent study.	4.05 ± 0.87	4.29 ± 0.74	0.048
3	I was able to summarize the key points of the outcome of the group discussion.	3.91 ± 0.90	3.99 ± 1.00	0.476
4	I managed my independent study effectively.	3.95 ± 0.92	4.23 ± 0.86	0.026
5	My behaviour encouraged other members to actively participate in the tutorial process.	3.67 ± 1.03	3.95 ± 1.01	0.024
6	I reflected on my learning in each scenario based on the objectives that I set myself.	3.98 ± 0.74	4.22 ± 0.86	0.046

**Fig. 2:**
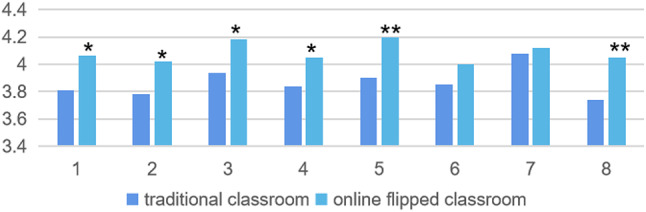
Comparison between online flipped classroom and traditional classroom in critical thinking. *Notes* *p < 0.05, **p < 0.005

2. In terms of students’ critical thinking, compared with the traditional classroom, the online flipped classroom has improved students’ critical thinking ability (Fig. 2), and several of them have improved significantly (Table 3): I analyzed information in the scenario using relevant theory and concepts (4.2 ± 0.74; *p* < 0.005), I could generate a discussion to explain the problem under discussion (4.05 ± 0.87; *p* < 0.005), I communicated my ideas clearly. (4.02 ± 0.94;*p* < 0.05), In the second meeting, I applied knowledge from my independent study to provide a solution to the problem being discussed(4.05 ± 0.84;*p* < 0.05).

**Table 3:** Comparison of active learning between online flipped classroom (*n* = 106) and traditional classroom (*n* = 172)

No	Items	Traditional classroom mean ± SD	Flipped classroom mean ± SD	*p* value
1	I was able to formulate questions based on the scenario.	3.81 ± 0.91	4.06 ± 0.93	0.042
2	I communicated my ideas clearly.	3.78 ± 1.03	4.02 ± 0.94	0.046
3	I performed the role given to me by other group members.	3.94 ± 0.93	4.18 ± 0.93	0.012
4	In the second meeting, I applied knowledge from my independent study to provide a solution to the problem being discussed.	3.84 ± 0.88	4.05 ± 0.84	0.049
5	I analysed information in the scenario using relevant theory and concepts.	3.9 ± 0.78	4.2 ± 0.74	0.002
6	I made links during the tutorial process between my newly acquired knowledge and my previous knowledge.	3.85 ± 0.92	4 ± 0.92	0.2
7	I explained knowledge from the resources in my own words.	4.08 ± 0.77	4.12 ± 0.80	0.634
8	I could generate a hypothesis to explain the problem under discussion.	3.74 ± 0.85	4.05 ± 0.87	0.004

3. In terms of satisfaction survey, we sent out questionnaires to students to find out whether they are satisfied with the online flipping class. The overall questionnaire adopts a five-level scoring system, and the answer options are scored from low to high as 1, 2, 3, 4, and 5, which are very dissatisfied, dissatisfied, general, satisfied, and very satisfied (Table 4). 105 questionnaires were collected. Among them, the overall satisfaction of students is 3.96 on average, and the rate of very satisfied or satisfied with the online flipped classroom teaching is 75.6%, and the answer value of each item is also high. The score of online flipped classroom encouraging students to learn more about the conditions discussed through cases is 4.11, and the score of 4.13 in more chapters that hope this teaching method will be used is the highest, It shows that learners are relatively satisfied with the online flipped classroom teaching mode and affirm the possibility of continuous development. In addition, many students think that the online flipping class plays a great role in improving self-learning ability, analysis ability and learning interest. At the same time, students are also satisfied with the design of the case, the knowledge covered by the case and the organization of the course.

**Table 4:** Analysis of students’ satisfaction with online flipped classroom (*n* = 105)

summary of students opinion	Strongly agree	agree	neutral	disagree	strongly disagree	mean ± SD
1. The learning style was effective in helping develop my self-directed learning skills	34.31%	41.18%	13.73%	7.84%	2.94%	3.96 ± 1.03
2. The learning style was effective in helping increase my problem-solving skills	29.41%	39.22%	17.65%	10.78%	2.94%	3.81 ± 1.07
3. The learning style was effective in helping sharpen my analytical skills	31.38%	43.14%	10.78%	11.76%	2.94%	3.88 ± 1.07
4. The learning style was effective in helping improve my expression/communication skills	33.33%	35.29%	15.69%	12.75%	2.94%	3.83 ± 1.12
5. The learning style was effective in helping increase my interaction/collaboration skills	33.34%	37.25%	17.65%	7.84%	3.92%	3.88 ± 1.08
6. The learning style greatly impacts my way of learning	37.26%	40.20%	10.78%	8.82%	2.94%	4 ± 1.05
7. The learning style motivated and inspired my interest to learn	33.33%	39.22%	15.69%	5.88%	5.88%	3.88 ± 1.12
8. The learning style improved my confidence in future patient encounters	37.26%	36.27%	12.75%	7.84%	5.88%	3.91 ± 1.16
9. Cases were well designed	34.31%	41.18%	18.63%	4.90%	0.98%	4.03 ± 0.91
10. Cases demonstrate appropriate knowledge of the subject area	31.37%	49.02%	13.73%	3.92%	1.96%	4.04 ± 0.89
11. Cases are relevant to real-world clinical situations	36.28%	44.12%	11.76%	3.92%	3.92%	4.05 ± 1
12. Using the cases has encouraged me to learn more about the discussed condition	33.34%	52.94%	7.84%	2.94%	2.94%	4.11 ± 0.89
13. The course was well organized in a way that helped me achieve the learning objectives and outcomes	34.31%	42.16%	15.69%	4.90%	2.94%	4 ± 0.99
14.The course created a positive learning environment where I felt supported in my learning	29.41%	43.14%	16.67%	7.84%	2.94%	3.88 ± 1.02
15. The learning style can be used in more chapters	37.25%	45.10%	12.75%	2.94%	1.96%	4.13 ± 0.89
16. Overall, I was satisfied with the quality of this course	35.30%	39.22%	10.78%	10.78%	3.92%	3.91 ± 1.12

## Discussion

In the teaching process of international students, the vast majority of international students show the characteristics of publicity and participation. Many teachers find that international students don’t like traditional teaching methods. They like to communicate and interact with teachers in class. Group discussion and other forms of teaching are also preferred by students. They believe that this can show students’ own learning ability and level. In addition, they have a strong desire to complete their studies and get a degree. Therefore, in the teaching process, we should reasonably mobilize and give full play to the positive factors and personality publicity characteristics of international students in learning, promote teaching reform, and promote students’ participation in the teaching process.

At present, the education of international students has become an important part of higher education. Most international students choose to return to work after graduation and need to take the local medical practitioner examination. However, the curriculum and teaching management mode of our school mostly follow Chinese standards and characteristics, and there is a certain disconnect with international medical standards, which makes it difficult for international students to adapt to the domestic examination mode in a short time after graduation, and often requires a lot of energy and time to prepare for the examination. The growth and success of overseas students in China has become an important way for the world to know and understand China, and is of great positive significance in establishing China’s international image and enhancing the friendship between the Chinese people and the people of all countries in the world. Therefore, we must actively reform the curriculum construction of international medical students in China to realize the international integration of international student education.

Flipped classroom (FC) refers to a teaching mode that flips the traditional classroom teaching (TC), through the use of advanced information technology, students learn by themselves through online videos recorded by teachers and learning requirements before class, so that students can complete the learning of knowledge before class, solve problems through teachers’ guidance, discussion and communication in class, promote the internalization of knowledge, and complete the evaluation of learning results and knowledge improvement after class.

In Morton DA et al. research, they wanted to determine whether FC instruction is superior to TC instruction for learning gross anatomy. The results showed that students in an FC setting may perform better than those in a TC on assessments requiring higher cognition (e.g., analysis), but the same on those requiring lower cognition (e.g., memorization and recall) [27]. To compare the effects of Flipped Classroom vs. Traditional Classroom on students’ academic achievement, task value, and achievement emotions, O’Connor EE et al. proved that the positive emotional effects of FC on medical students’ motivational beliefs and achievement emotions can enhance academic performance. The FC approach provides medical students with the opportunity to develop self-directed learning skills while also providing opportunities to solidify already acquired knowledge and concepts through active learning strategies [28]. At last, in the research of Kraut et al. a total of 54 papers (33 quantitative, four qualitative, and 17 review) on FC met a priori criteria for inclusion and were critically appraised and reviewed. The top 10 highest scoring articles (five quantitative studies, two qualitative studies, and three review papers) are summarized in this article. Their results confirmed that (1) A Flipped Classroom or Blended Learning Approach is Effective for Procedural Learning; (2) Students in a Flipped Classroom Setting May Learn More Than Students in a Traditional Classroom Setting; (3) The Flipped Classroom Model is Beneficial for Learning Higher Cognition Tasks; (4) Learners Are More Engaged with Flipped Classroom, but Satisfaction Depends Largely on Teacher Prep Work [29].Therefore, flipped classroom can improve the purpose of teachers’ teaching, endow international students with autonomy in learning, and enhance the teaching effect. It has become a new concept and teaching mode of international students’ education and teaching in the stimulating the learning motivation and professional interest of international students, enrich teaching means, enhance teaching attraction, and increase teaching interaction; At the same time, by strengthening the evaluation of in class and online learning of international students, we can increase their active learning ability, promote personalized learning, improve their autonomous learning ability and critical thinking ability, promote the development of pathophysiology course, and integrate with international medical education.

Several studies showed that flipped classroom combined with case-based learning is an effective teaching modality in nephrology clerkship and other medical class [30, 31]. Cai et al. also proved that CBL-based FC modality has promising effects on undergraduate pathology education and may be a better choice than traditional LBC. Further optimizations are needed to implement this novel approach in pathology and other medicine curricula [32]. Various evidence-based and student-centered strategies such as Team-Based Learning (TBL), Case-Based Learning (CBL), and Flipped Classroom (FC) have been recently applied to anatomy education and have shown to improve student engagement and interaction [33, 34].

The advisability of early contact with clinical knowledge has been accepted by the world. There is no better teacher than “interest”. Let medical students contact clinical knowledge as early as possible, and closely combine book knowledge with clinical knowledge, which will improve students’ learning enthusiasm and cultivate good habits: active learning, autonomous learning, combined with clinical practice, so that students can give play to their subjective initiative.

Pathophysiology, as a bridge discipline between basic medicine and clinical medicine, plays a connecting role in medical education. Through the study of pathophysiology, students are trained to understand and deal with the scientific thinking methods of diseases, grasp the leading links and development trends of diseases, correctly use the knowledge and theory of pathophysiology, understand the essence of diseases from the outside to the inside, analyze and discuss the causes, mechanisms, functional metabolic changes and prevention and treatment principles of diseases, so as to cultivate students’ independent active learning ability, critical thinking ability and clinical thinking ability. Therefore, we adopt the teaching mode of “CBL + FC” in the teaching process. It is hoped that through the reform of this teaching mode, the autonomous learning ability and critical thinking ability of international students will be improved, and the learning of clinical cases will be used to realize the cultivation of early clinical thinking.

The results of this study show that compared with the traditional classroom, the online flipped classroom based on CBL has significantly improved the learning enthusiasm and enthusiasm of international students. Through independent literature review, PPT production, active participation in classroom teaching, learning to use a variety of learning strategies, and cooperation with other students. At the same time, the students’ ability to raise problems and solve problems has been greatly improved by analyzing clinical cases; By consulting the literature, the theoretical knowledge learned can be better applied to clinical analysis. The results of the satisfaction survey also show that international students are more likely to accept the flipped classroom teaching mode. Therefore, in view of the characteristics and needs of the international students in our university, the teaching reform of Pathophysiology adopts CBL + FC, focusing on stimulating the learning motivation and professional interests of the international students. By strengthening the evaluation of the internal and external and online learning of the international students, we can increase the independent learning ability, improve the critical thinking ability and promote personalized learning, which will be conducive to the development of the Pathophysiology course and integrate with international medical education.

This study had some limitations. Firstly, the international students’ sample size was limited, which might impact on the power of these results. Secondly, due to the impact of the epidemic, flipped classes cannot be carried out offline and face-to-face with students, which will also affect the effect of teaching reform. However, the overall effectiveness of CBL + FC can be enhanced by leveraging the unique characteristics of international students and promoting their enthusiasm for learning. Continuous evaluation and adaptation of teaching strategies are essential to ensure that all students benefit from the learning process and that the educational needs of international students are met effectively.

## Conclusion

Through the training of this project, the students will not only have solid basic theoretical knowledge, but also have good communication ability and preliminary clinical thinking ability.

## Data Availability

Datasets generated or analyzed in this study are included in this published article. The Stata raw dataset can be provided on request. The corresponding author, Fang Tian, will provide additional data, if requested.
